# IRIS—Intelligent Rapid Interactive Segmentation for Measuring Liver Cyst Volumes in Autosomal Dominant Polycystic Kidney Disease

**DOI:** 10.3390/tomography8010037

**Published:** 2022-02-09

**Authors:** Collin Li, Dominick Romano, Sophie J. Wang, Hang Zhang, Martin R. Prince, Yi Wang

**Affiliations:** Department of Radiology, MRI Research Institute, Weill Cornell Medicine, New York, NY 10065, USA; cli5@student.gn.k12.ny.us (C.L.); djr327@cornell.edu (D.R.); swang50@stuy.edu (S.J.W.); hz459@cornell.edu (H.Z.); map2008@med.cornell.edu (M.R.P.)

**Keywords:** liver cyst, lesion segmentation, intelligent rapid interactive segmentation

## Abstract

Purpose: To develop and integrate interactive features with automatic methods for accurate liver cyst segmentation in patients with autosomal dominant polycystic kidney and liver disease (ADPKD). Methods: SmartClick and antiSmartClick were developed using iterative region growth guided by spatial and intensity connections and were integrated with automated level set (LS) segmentation and graphical user interface, forming an intelligent rapid interactive segmentation (IRIS) tool. IRIS and LS segmentations of liver cysts on $T_{2}$ weighted images of patients with ADPKD (*n* = 17) were compared with manual segmentation as ground truth (GT). Results: Compared to manual GT, IRIS reduced the segmentation time by more than 10-fold. Compared to automated LS, IRIS reduced the mean liver cyst volume error from 42.22% to 13.44% (*p* < 0.001). IRIS segmentation agreed well with manual GT (79% dice score and 99% intraclass correlation coefficient). Conclusion: IRIS is feasible for fast, accurate liver cyst segmentation in patients with ADPKD.

## 1. Introduction

Most patients with autosomal dominant polycystic kidney disease (ADPKD) develop polycystic liver disease in their later life [1]. Liver cyst volume in ADPKD is associated with decrement in quality of life and morbidity and is an important biomarker for clinical decision-making, including cyst fenestration, partial liver resection, and liver transplantation [1,2,3]. Liver cysts in ADPKD are hyperintense on $T_{2}$ weighted MRI and can be readily measured with standard tools available on picture archival computer systems (PACS) when there are only a few cysts. However, many ADPKD patients, and especially females, have hundreds of cysts which are challenging to measure manually. Since manual lesion segmentations are very tedious and time-consuming, cyst volume is often approximated by radiologists’ impression without segmentation which has interobserver variability and limited accuracy. Manual segmentation can be improved with various interactive segmentation methods that use various user inputs, such as partial segmentation, as initialization to an automated output; however, these techniques are all restrictive and tedious [4]. Automated volume measurement of numerous and heterogeneous cysts is needed for ADPKD patients; however, it is difficult to achieve [5,6].

The purpose of this study is to develop a rapid semiautomated lesion segmentation tool combining rapid interactive edits with automated segmentation based on computer vision techniques: intelligent rapid interactive segmentation (IRIS). The liver cyst labeling process is intelligently automated while allowing for user interaction to rapidly improve the automated segmentation. We evaluated the speed and accuracy of IRIS for fast liver cyst segmentation in 17 patients with ADPKD.

## 2. Methods and Materials

### 2.1. Intelligent Rapid Interactive Segmentation (IRIS) Method and Implementation

Image data were preprocessed for the liver region of interest (ROI) segmentation preprocessing using a convolutional neural network with residual layers [7], and image intensity was scaled to [0, 1] using a min–max normalization. First, automatic level set (LS) cyst segmentation is performed in the liver ROI using the Chan–Vese level set algorithm [8]. The locations of liver cysts on each axial slice within the liver ROI were determined using a small region of the lowest voxel intensity (typically the right anterior corner) as an initial level set function.

IRIS provided a graphic user interface for editing the automated LS segmentation with smart features, SmartClick and antiSmartClick, in addition to a simple paintbrush. Similar to the recursive region growing guided by intensity similarity for segmenting the left ventricle in cardiac MRI [9,10], SmartClick determined a targeted new voxel in a liver cyst for growth (*g*) at a given iteration by thresholding over an affinity sensitivity relative to its neighboring seed voxel (*s*) according to their intensity (*I*) difference, $\rho \left(g,s\right)=(1-{\left|I\left(g\right)-I\left(s\right)\right|}^{a})$, where a= adjustable sensitivity [4]. This recursive region growth started with a click-defined seed point, and the growth stopped when the affinity sensitivity was under the set threshold (*p* < 0.5). On the other hand, the antiSmartClick employed the same feature as SmartClick but recursively removed voxels for erasing erroneously included voxels in segmentation such as $T_{2}$ bright vessels or bile ducts. To allow addressing voxel intensity heterogeneity within a liver cyst, SmartClick included a closing operation of dilation and erosion to fill holes within a lesion.

### 2.2. Patient Population

MRI data were obtained from patients enrolled in the Polycystic Kidney Disease Repository (NCT00792155, https://clinicaltrials.gov/ct2/show/NCT00792155, accessed on 31 January 2022), an ongoing longitudinal investigation of ADPKD conducted at our medical center [11]. The scans sampled in this study were collected on 1.5 T (*n* = 5) and 3 T (*n* = 12) MRI systems (General Electric, Milwaukee, WI, USA; Siemens, Erlagen, Germany) between 1 January 2017 and 31 July 2021. Cases were selected randomly from ADPKD-positive subjects, and selection was based on the presence of $T_{2}$ hyperintense liver cysts throughout the liver region of interest (ROI) on $T_{2}$ weighted (T2w) MR images. Typically, 72 (ranging from 32 to 100) axial T2w images were acquired using a single-shot fast spin echo sequence with breath-holding to cover the whole liver. This retrospective analysis of existing patient images was approved by the local institutional review board, and all images were deidentified prior to liver cyst segmentation.

### 2.3. Data Analysis

The performance of IRIS for liver cyst segmentation in ADPKD was evaluated by comparing with manual reference ground truth (GT), as well as the automated level set (LS). Segmentation times were recorded for all methods. The similarity between the segmented cyst spatial distributions by the two methods was evaluated using the dice score to assess geometric match and intraclass correlation coefficient (ICC) to assess volume agreement. The liver cyst signal-to-noise ratio (SNR) was measured as the cyst signal intensity ROI mean over standard deviation; the liver cyst–liver contrast-to-noise ratio (CNR) was measured as the cyst to adjacent liver signal difference over the cyst signal ROI standard deviation; and the cyst SNR and CNR measurements were performed over three representative cysts per patient. The 1.5 T cases were compared with 3 T cases with matching cyst patterns. The liver cyst volumes measured on all segmentations were compared using linear regression and the Bland–Altman plot. The statistical significance between LS and IRIS was assessed using Student’s *t*-test.

## 3. Results

For 3 T vs 1.5 T matched comparison, the cyst SNR was 42.45 ± 25.20 vs. 19.74 ± 14.40 (*p* = 0.05), the cyst CNR was 3.26 ± 0.95 vs. 2.64 ± 0.81 (*p* = 0.25), the manual processing time was 2072 ± 1383 vs. 2369 ± 925 (*p* = 0.64), and the IRIS processing time was 179 ± 72 vs. 236 m ± 82 (*p* = 0.26). There was no significant difference in processing time between 1.5 T and 3 T; for further analysis, all cases were aggregated together and the cyst SNR/CNR on average over all cases was 35.77/3.08.

**Figure 1:**
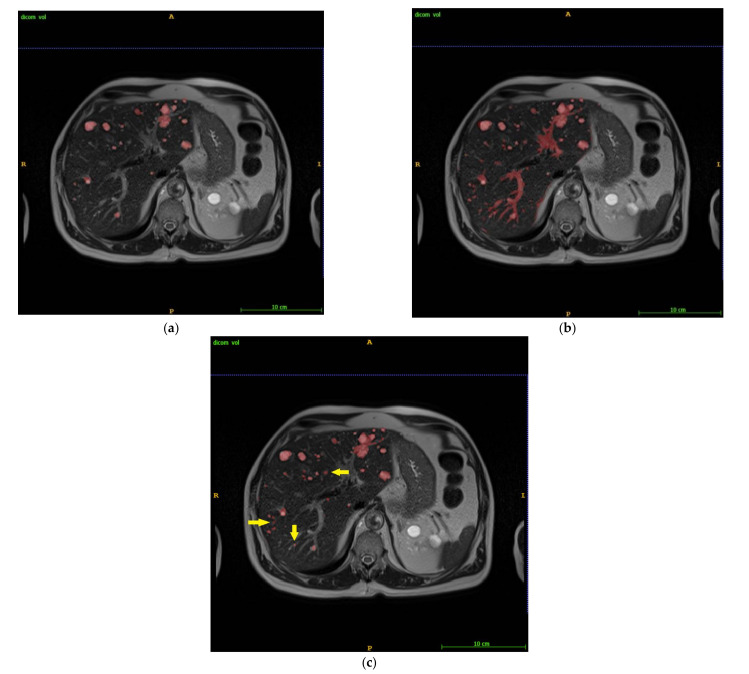
Liver cyst segmentation by (**a**) manual as ground truth (GT), (**b**) automated level set (LS), and (**c**) IRIS. LS included substantial vasculature (**b**), which was cleaned up rapidly using antiSmartClick (**c**). Small cysts missed on manual GT were captured on IRIS (arrows in (**c**)).

Figure 1 illustrates an example of manual liver cyst segmentation by manual GT (Figure 1a), automated LS segmentation (Figure 1b), and IRIS (Figure 1c). The substantial LS error of including vasculature and bile ducts was rapidly removed using antiSmartClick (Figure 1b vs. Figure 1c). There were small cysts missed in manual GT but captured on IRIS (arrows in Figure 1c).

Speed metrics comparing GT, LS, and IRIS are shown in Table 1. On average, the IRIS method took 202 s, which was 10.7 times faster than manual GT of 2159 s (*p* = 8.92 × 10^−6^).

**Figure 2:**
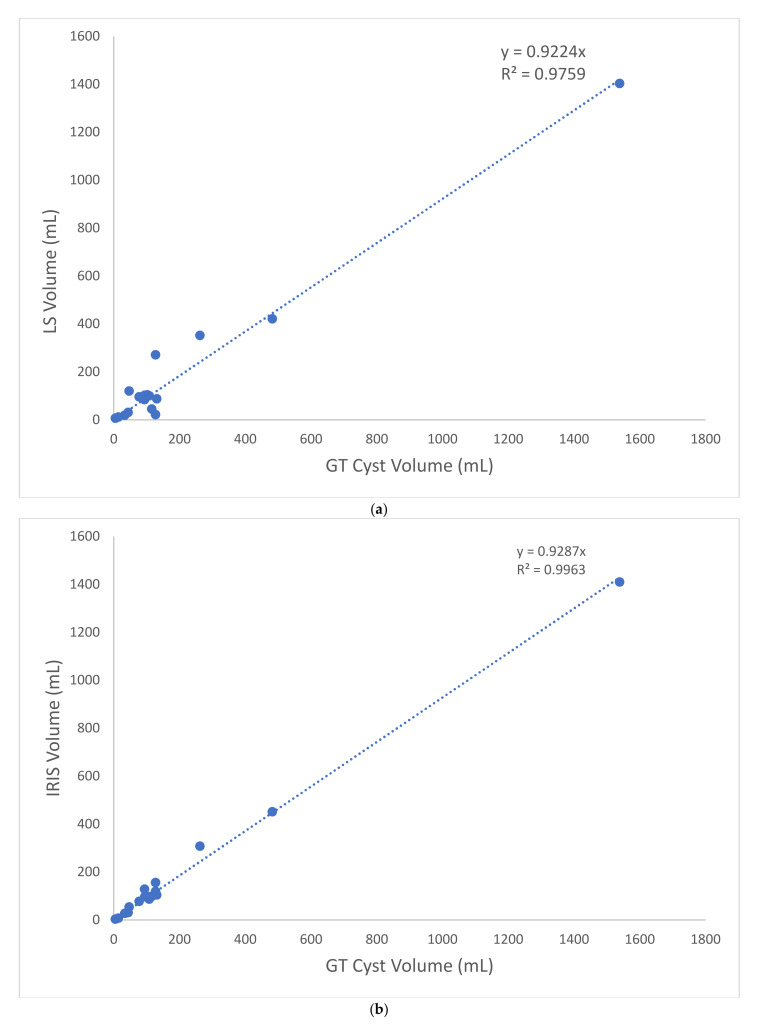
Scatter plots of liver cyst volume measurements by (**a**) LS and (**b**) IRIS against GT. IRIS improved regression slope and regression coefficient. GT = ground truth segmentation, LS = automatic level set segmentation, and IRIS = intelligent rapid interactive segmentation.

**Figure 3:**
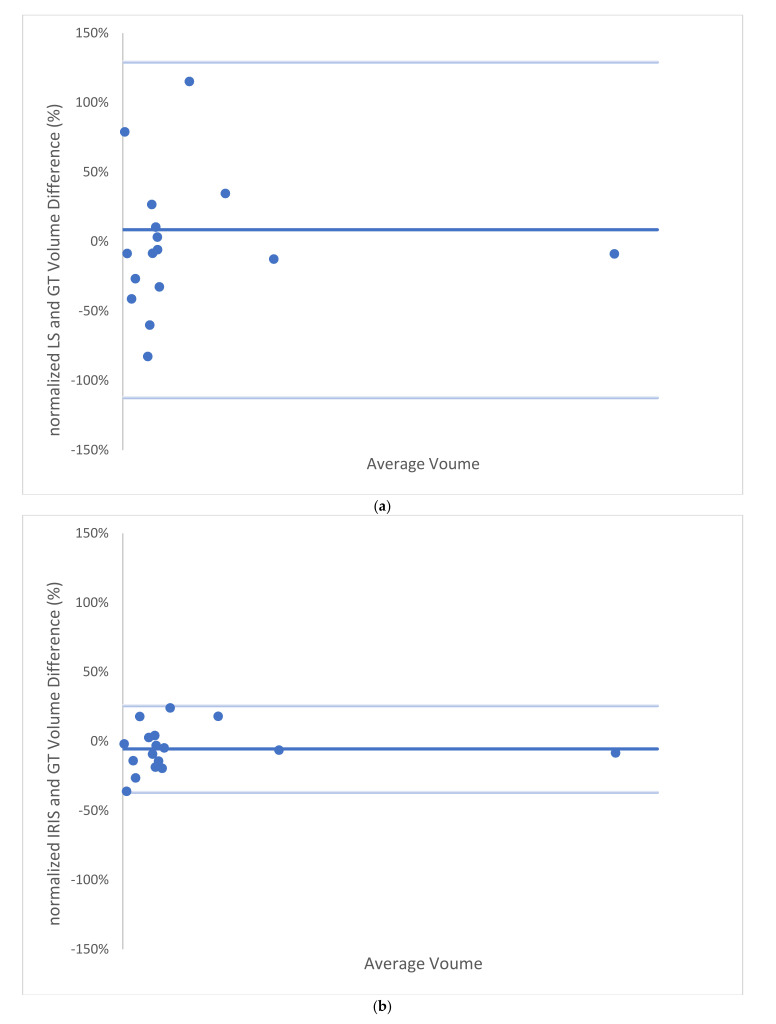
Bland–Altman plots for (**a**) LS and (**b**) IRIS for cyst volume measurements. LS had a bias of 8.53%, STD of 61.61%, lower limit of agreement (LLA) of −112%, and upper limit of agreement (ULA) of 129%. (**a**). IRIS had a bias of −5.49%, STD of 15.94%, LLA of −37%, and ULA of 26%. GT = ground truth segmentation, LS = automatic level set segmentation, IRIS = intelligent rapid interactive segmentation, and STD = standard deviation.

Accuracy metrics comparing GT, LS, and IRIS are shown in Table 2, Table 3 and Table 4 and Figure 2 and Figure 3. According to Table 2, Table 3 and Table 4, for comparing to LS, IRIS increased the mean ± standard deviation (STD) dice score from 63.1 ± 18.0% to 79 ± 9.2% (Table 2, *p* = 5.53× 10^−4^), decreased the mean ± STD liver cyst volume (error normalized by GT volume) from 42.22 ± 44.49% to 13.44 ± 9.70% (Table 3, *p* = 0.0097), increased ICC from 98.91% to 99.54%, and decreased the liver cyst volume root mean squared error (RMSE) from 66.4 mL to 35.9 mL (Table 4).

**Figure 4:**
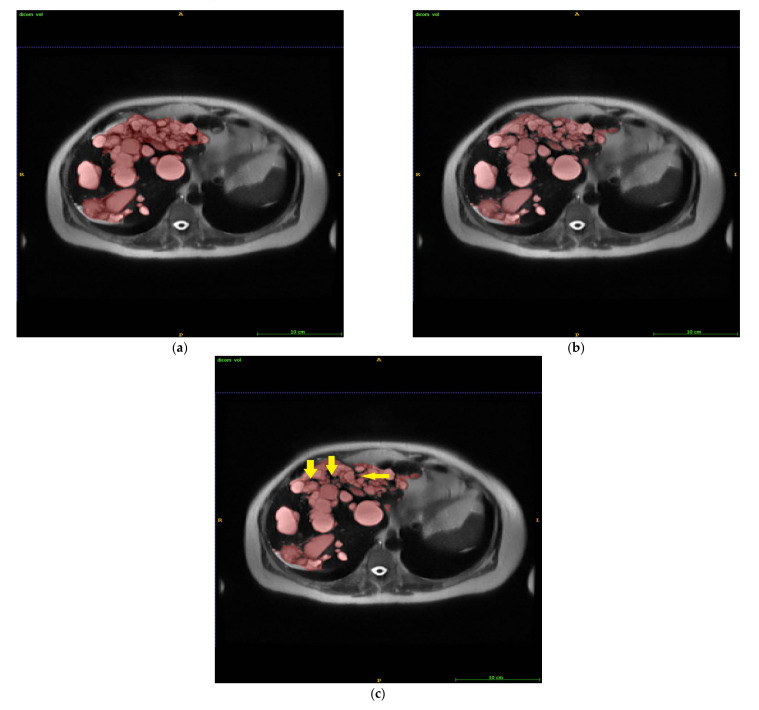
Liver cyst segmentation by manual GT (**a**), automated LS (**b**), and IRIS (**c**). The voxels in the space between neighboring cysts (arrows in (**c**)) were easily included in the manual GT, contributing to the observed segmentation errors. GT = ground truth segmentation, LS = automatic level set segmentation, and IRIS = intelligent rapid interactive segmentation.

According to Figure 2 and Figure 4 for comparing to LS, IRIS increased linear regression slope/coefficient (R^2^) from 0.922/0.976 (Figure 2) to 0.929/0.996 (Figure 3), and reduced bias/[lower, upper] limit of agreement from 8.53%/[−112, 129]% to −5.49%/[−37, 26]% (Figure 3).

The discrepancies between manual GT, automated LS, and IRIS segmentations were reviewed and were largely caused by voxels at cyst edges and/or in spaces between neighboring cysts that were easily included in manual segmentation, as illustrated by arrows in Figure 4 and also subtly noticeable on Figure 1.

## 4. Discussion

Our preliminary results demonstrate the feasibility of IRIS for fast and accurate segmentation of liver cysts in patients with ADPKD. The SmartClick and antiSmartClick features in IRIS allow rapid edits of automated level set (LS) segmentation, significantly improving cyst segmentation accuracy as measured by dice score and liver cyst volume differences. IRIS reduces the liver cyst segmentation time by more than 10-fold compared to manual segmentation, down to 3.4 min on average, making segmentation of numerous cysts in ADPKD liver clinically feasible.

The large number and heterogeneity of liver cysts in ADPKD patients have made automated segmentation of these cysts challenging [5,6]. A major cause of failure for automated segmentation of numerous lesions in ADPKD is the signal intensity variation across the liver volume [6]. The interactive features in IRIS address this unreliability by clicking into a small region where intensity variation within a cyst is small and segmentation can be robustly and rapidly performed using one click, as we have learned from segmenting the bright left ventricle from the surrounding dark myocardium in cardiac MRI [9,10]. The SmartClick and antiSmartClick in IRIS are based on iterative region growth guided by spatial and intensity connections, which has been shown to be highly robust in cardiac MRI [9,10]. Our data here demonstrate that SmartClick and antiSmartClick, originally developed for left ventricle segmentation, can be effectively applied to liver cyst segmentation. SmartClick and antiSmartClick can be incorporated into any interactive viewing program to edit and ensure the accuracy of any automated lesion segmentation, including those based on deep learning [12,13].

The level set method selected for automated cyst segmentation in this study is a popular approach that starts with the user’s initial segmentation and evolves into the desired segmentation [14]. It is known that the relationship between the initial border and the final segmentation of the level set is problematic. Consequently, the level set segmentation threshold may cause segmentation flooding into nearby organs or background tissues with similar voxel intensities. Here, cropping of adjacent organs and tissues by starting with an initial liver ROI segmentation effectively resolved the issue [14]. Deep learning is another approach for automated lesion segmentation [15,16], which is becoming increasingly more popular [13]. For segmenting the numerous cysts in an ADPKD liver, there is no labeled data for training a deep neural network (DNN). IRIS can be used to rapidly curate cyst labels for training DNN and extend the deep learning used in liver ROI segmentation in this work into liver cyst segmentation.

It should be noted that manual segmentation is not a perfect ground truth. Small cysts can be missed on manual segmentation because it is so tedious and tiring, as exemplified in Figure 1. The largest normalized volume error (38.7%) occurred in a case with a small liver cyst volume (13 mL by manual segmentation and 8 mL by IRIS) and was largely caused by discrepancies at border voxels. The large absolute volume error (35.9 mL) occurred in a case with a large liver cyst volume (1538 mL by manual and 1411 mL by IRIS) and was largely caused by voxels in spaces between neighboring cysts (Figure 4). We feel that IRIS can provide a more consistent border definition for cysts and, therefore, a better ground truth.

This encouraging preliminary study has identified several limitations for future improvements. (1) Both manual and level set segmentation have difficulties in segmenting small cysts due to poor conspicuities, which remains a challenge for IRIS. Though the current clinical management is based on total liver cyst volume, small cyst identification may allow the study of their progression. A cause of poor conspicuity is motion artifacts, which should be minimized as required so in clinical practice. Another case of poor conspicuity is noise, which should be further investigated. (2) Vascular structures also appear hyperintense on $T_{2}$ weighted images and are commonly included in the level set, which is the major requirement of IRIS editing. Future automated segmentation development, such as using deep learning or constraining labels into circular or spherical geometries, may reduce vasculature/biliary editing and further speed up IRIS. Current data acquisition of thick slices through the liver volume makes it difficult to differentiate vascular structures that are connected and extended in space from cysts that are approximately spherical. Higher-resolution volumetric imaging with respiratory motion compensation [17,18,19,20,21,22,23,24] may help with accurate liver cyst volume measurement and to overcome the potential slice misregistration from multiple breath-holds that affect cyst volume measurements. (3) The partial volume effect in voxels of mixed cyst and normal liver tissue may need to be accounted for, particularly in images with large voxels or thick slices, by geometric and biophysical modeling [10,25]. (4) It is desired to minimize human interaction in segmentation, as human interaction can introduce error and operator variability. Deep learning discussed above may be integrated with IRIS for continuous training and improvement to minimize human interaction in future accurate liver cyst segmentations.

## 5. Conclusions

In summary, intelligent rapid interactive segmentation (IRIS) is feasible for fast and accurate liver cyst segmentation in autosomal dominant polycystic kidney disease (ADPKD), using SmartClick and antiSmartClick to rapidly refine automated level set segmentation. The accuracy performance of IRIS segmentation closely matches that of the manual segmentation.

## Figures and Tables

**Table 1:** Compared segmentation times among manual segmentation ground truth segmentation (GT), automatic level set segmentation (LS), and intelligent rapid interactive segmentation (IRIS). STD = standard deviation.

Time (s)	GT	LS	IRIS
Mean	2159	6	202
Median	2145	5	187
[Min, Max]	[293, 5427]	[2, 28]	[75, 338]
STD	1312	6	84

**Table 2:** Compared dice scores of LS and IRIS against GT. GT = ground truth segmentation, LS = automatic level set segmentation, IRIS = intelligent rapid interactive segmentation, and STD = standard deviation.

Dice Score	GT—LS	GT—IRIS
Mean	63.1%	79.0%
Median	63.8%	81.0%
[Min, Max]	[29.7%, 90.5%]	[58.2%, 93.5%]
STD	18.0%	9.2%

**Table 3:** Compared automatic LS segmented cyst volume (V_LS_) and IRIS segmented cyst volume (V_IRIS_) against manual GT segmented cyst volume (V_GT_). GT = ground truth segmentation, LS = automatic level set segmentation, and IRIS = intelligent rapid interactive segmentation.

Metric	|V_GT_−V_LS_|/V_GT_	|V_GT_−V_IRIS_|/V_GT_
Mean	42.22%	13.44%
Median	26.84%	13.87%
[Min, Max]	[3.34%, 161.58%]	[1.74%, 35.90%]
STD	44.49%	9.70%

**Table 4:** ICC and RMSE of LS and IRIS segmentation against GT for cyst volume measurement. GT = ground truth segmentation, LS = automatic level set segmentation, IRIS = intelligent rapid interactive segmentation, ICC = intraclass correlation coefficient, and RMSE = root mean squared error.

Metric	LS	IRIS
ICC	98.91%	99.54%
RMSE	66.4 mL	35.9 mL

## Data Availability

The data presented in this study are available upon reasonable request to the corresponding author. The data are not publicly available due to the sensitive nature of medical image data.
